# Fecal metabolome alterations in infants at risk of developing allergies during the first year of life

**DOI:** 10.1007/s11306-026-02478-6

**Published:** 2026-07-01

**Authors:** Mariyana V. Savova, Pingping Zhu, Alida Kindt, the TEMPO study team, Harm Wopereis, Clara Belzer, Amy C. Harms, Thomas Hankemeier

**Affiliations:** 1 Metabolomics and Analytics Centre, Leiden Academic Centre for Drug Research, Leiden University, 2333 CC Leiden, the Netherlands; 2 Danone Research & Innovation, Uppsalalaan 12, 3584 CT Utrecht, the Netherlands; 3 Laboratory of Microbiology, Wageningen University, Stippeneng 4, 6708 WE Wageningen, the Netherlands

**Keywords:** Early life, Birth mode, Stool metabolomics, Solid food introduction, Allergy, LC-MS

## Abstract

**Introduction:**

Disturbances in the infant gut microbiome (GM) may increase the risk of developing allergies. This critical developmental period is characterized by rapid microbial colonization, which is influenced by factors like delivery mode and infant feeding practices.

**Objectives:**

The present study investigated changes in key GM taxa and fecal metabolites in relation to allergy development, delivery mode, age, and infant feeding practices during the first year of life.

**Methods:**

Seventy-two infants at risk of allergies, exclusively breastfed for at least 16 weeks, were followed in their first year. During this period, allergy manifestations were recorded and fecal samples collected at three time points. The samples were subjected to metabolic profiling covering host and microbial metabolites and fluorescent in situ hybridization to quantify *Bifidobacterium* spp. and the *Eubacterium rectale/Clostridium coccoides* group.

**Results:**

Strong age-associated metabolic shifts were observed, particularly in aromatic amino acid metabolites, bile acids, B vitamins, and short and long-chain fatty acids. Feeding practices, specifically the introduction of complementary feeding and the cessation of breastfeeding were significantly associated with changes to the fecal metabolome. Delivery mode had a pronounced impact on the metabolome, with differences between vaginal and Cesarean deliveries persisting until 6 months of age. Infants who developed an allergy during this period had lower *Bifidobacterium* spp. and significantly higher polyunsaturated fatty acid levels before the age of 16 weeks.

**Conclusion:**

This study offers valuable insights into the longitudinal development of the fecal metabolome and factors influencing it during infancy, a critical period for immune system development.

**Clinical trial registration:**

Clinicaltrials.gov identifier: NCT03067714, registered: 01/02/2017.

**Supplementary Information:**

The online version contains supplementary material available at https://doi.org/10.1007/s11306-026-02478-6.

## Introduction

Our guts are home to trillions of microorganisms, including bacteria, archaea, viruses, and fungi, that live in a symbiotic relationship with us as hosts (Vemuri et al., 2020; Wu & Wang, 2019). The first year of life is crucial for the development and maturation of the gut microbiome (GM) (Yatsunenko et al., 2012). Early‑life GM development is characterized by a transition from an infant‑type microbiota dominated by *Bifidobacterium* spp. to a more diverse, adult‑like community enriched in obligate anaerobes such as members of the *Lachnospiraceae* (Laursen et al., 2016). This period also coincides with the development of the immune system (Simon et al., 2015) and is a key window in which GM colonization shapes the host’s immune system (Gensollen et al., 2016). An accumulating body of research links the disturbances of the GM composition in early life to a multitude of immune-mediated diseases, (Sarkar et al., 2021) including allergies (Fazlollahi et al., 2018; Savova et al., 2024). Allergic disease often follows a temporal progression from atopic dermatitis and food allergy in infancy to allergic asthma and rhinitis in childhood, also known as “atopic march” (Yang et al., 2020). The study of early life GM composition and function in relation to allergy development is therefore a topic of considerable interest.

Many factors are known to influence the GM composition in infancy, including use of antibiotics, mode of delivery (vaginal versus C-section), milk feeding practices (breastfeeding versus formula feeding), and the transition to solid foods (complementary feeding) (Milani et al., 2017). The use of antibiotics and C-section have been associated with dysbiosis in early life and risk of developing of atopic dermatitis and other diseases later in life (Hoskinson et al., 2024; Ríos-Covian et al., 2021). Even though the effect of breastfeeding on allergy is still a topic of debate, breastfeeding is the recommended infant nutrition for allergy prevention (Nuzzi et al., 2021). Breast milk is considered the optimal nutrition for infants due to its balanced composition of macronutrients and bioactive compounds satisfying the infant’s nutritional and physiological requirements (Garwolińska et al., 2018). It is also an important source of bifidobacteria and lactobacilli as well as human milk oligosaccharides (HMOs) (Parigi et al., 2015). Bifidobacteria, e.g. *B. breve*, *B. bifidum*, *B. longum*, capable of utilizing HMOs and derivatives for energy, thrive in the guts of healthy breastfed infants and are crucial for immune system development (Lin et al., 2022).

While the impact of the above-mentioned factors on the GM composition is relatively well-studied, their influence on GM activity remains understudied. Similarly, research examining the link between allergies and the GM have mainly focused on compositional analysis (Savova et al., 2024). Since the GM influences the host’s physiology via the production of metabolites, researchers are increasingly examining the metabolome to get insights into host-microbiota interactions (Krautkramer et al., 2021).

In this study, healthy breastfed infants at increased risk of developing allergies were followed during their first year. Data on delivery mode, allergy development, feeding practices were collected, and key gut microbial taxa along with the fecal metabolome were analyzed at three time points. Fluorescence in situ hybridization (FISH) was applied to quantify *Bifidobacterium* spp. and *Eubacterium rectale/Clostridium coccoides* (ER/CC) group, primarily composed of *Lachnospiraceae* species, as two abundant and functionally relevant microbial taxa. Unlike relative, sequencing-based approaches, FISH relies on direct bacterial cell enumeration, enabling robust assessment of temporal dynamics across infancy. Meanwhile, a combination of gas chromatography - flame ionization detector (GC-FID) and liquid chromatography-mass spectrometry (LC-MS) based fecal metabolomics was used to profile both host and microbial metabolites, including (aromatic) amino acids metabolites, B vitamins, bile acids, and short-chain fatty acids (SCFAs). This allowed us to assess microbiome and metabolomic changes associated with allergy, delivery mode, age, milk feeding practices (breastfeeding and formula feeding), and complementary feeding in infancy.

## Experimental section

### Study design, sample collection and storage

The samples for this work arise from a randomized, double-blind, controlled, parallel-group, multi-country study called TEMPO (clinicaltrials.gov identifier: NCT03067714). Detailed information on ethics committees, institutional review boards, and regulatory authorities that approved the study was previously published (Papadopoulos et al., 2022). The parent TEMPO study enrolled 855 healthy term infants (age: <16 weeks) at increased risk of developing allergy based on family history of allergy, defined as having at least one first-degree relative (mother, father, or sibling) with a documented allergic condition. An overview of baseline family allergy history is presented in Table S1. The 597 subjects who began formula feeding before 16 weeks were randomized to one of the two intervention arms. Of the remaining 258 infants, 232 met the criteria for exclusive breastfeeding for at least 16 weeks and comprised the breastfed reference group. Exclusive breastfeeding was defined as receiving only breast milk, with no other liquids or solids except water or formula in the first 72 h of life, disregarding vitamins, minerals, or medicines. All participants were followed for a year, during which events of allergic manifestations were diagnosed by qualified physicians and classified as skin, food, or respiratory allergies. Allergy manifestations were considered IgE-mediated if either the skin prick test to any tested allergen or specific IgE blood test was positive at 12mo. In this study, we selected a subset of 72 subjects solely from the breastfed reference group based on the availability of fecal samples collected before 16 weeks (baseline), at 6 months (6mo), and at 12 months (12mo) of age at the time of analysis. For schematic overview of the sample selection process refer to Figure S1. Fecal samples were collected at home and immediately stored in freezers, then transferred on ice to the participant hospitals and stored at − 80 °C until transfer to Danone Research & Innovation (Utrecht, The Netherlands) for wet sample aliquoting and fluorescence in situ hybridization (FISH), SCFAs and lactic acid analysis. Sample aliquots for LC-MS metabolomics analysis were transferred on dry ice to Leiden University and stored at − 80 °C until analysis.

### Quantification of *Bifidobacterium* genus and *Eubacterium rectale/Clostridium coccoides* group (ER/CC)

FISH quantification of *Bifidobacterium* genus and ER/CC was performed on a subset of subjects as described previously (Sim et al., 2012). Total bacteria count was determined using 4′,6-diamidino-2-phenylindole (DAPI) staining. This allowed us to express *Bifidobacterium* genus and ER/CC as proportion of the total bacterial count (Sim et al., 2012).

### Metabolomic data acquisition

LC-MS metabolomic data acquisition and preprocessing were performed as previously described (Zhu et al., 2025). Briefly, wet fecal samples went through lyophilization and liquid-liquid extraction prior to the analysis by reverse phase LC-MS (RPLC-MS) using two separate assays one covering polar to semi-polar metabolites and a second covering bile acids (BAs) and long-chain fatty acids (LCFAs). In case of coelution, the targets were reported using the name or abbreviation of one of the targets followed by a “#” (Table S2). Data quality inspection, including between-batch correction and removal of the 29 metabolites with high technical variance (quality control RSD > 30%) was conducted using mzQuality (Peet et al., 2025). The analysis of SCFAs and lactic acid was conducted as already described (Wopereis et al., 2018).

### Data analysis

Data handling and statistical analyses were performed in R (version 4.3.3). Information on the packages used is available in Supplementary Material. After dry weight normalization, metabolites with a median signal below five times the mean signal of the procedure blanks were excluded. To detect group bias in missing data, the Fisher’s exact test was applied to metabolites with any missing measurements (Tables S3-S4). The 57 metabolites with missingness > 20% were subjected to unpaired Mann-Whitney U test to assess the difference between visits and between the study groups (allergic vs. non-allergic, vaginal vs. C-section delivery, complementary-fed vs. non-complementary-fed, formula-fed vs. non-formula-fed, breastfed vs. non-breastfed) at the relevant visits. Following log_2_ transformation, missing values of the 162 metabolites with missingness < 20% were imputed using quantile regression imputation of left-censored data method (Wei et al., 2018). Then, linear mixed models (LMMs) were built to examine the metabolomic difference between the study groups over time using the lmerTest package (Kuznetsova et al., 2017). The allergic groups were also examined at baseline using a linear model. Details on the models used are available in Supplementary Material.

Clinical characteristics were checked for associations to allergy, delivery mode, and feeding practices using Mann-Whitney U-test for numeric variables and the Fisher’s exact test for binary variables. Differences in the microbiome data across visits and between the study groups at each visit were assessed using the Mann-Whitney U test. Spearman’s correlation analysis was conducted to assess the relationship between the microbiome taxa and metabolites found to associate with breastfeeding and allergy. Multiple testing correction was performed using the Benjamini-Hochberg method where Q < 0.1 was considered as statistically significant.

## Results

### Patient characteristics

Table 1 summarizes the characteristics of the 72 infants at risk of developing allergy who were followed throughout their first year. The associations between the clinical characteristics and allergy manifestation, delivery mode, and feeding practices, were examined (Tables S5-S7). Potential confounders not included in this analysis include: (i) clinical characteristics describing symptoms of allergy and its treatment, as well as gestational age and maternal pre-pregnancy BMI associated with c-sections; (ii) patient characteristics such as country and mineral supplementation which were excluded due to low sample size.

**Table 1:** Clinical characteristics

Variable	Whole cohort	Allergic	Non-allergic	C-section	Vaginal
Sex (female/male)	35/37	9/11	26/26	12/8	23/29
Allergy manifestation (allergic/not allergic)	20/52†	–	–	7/13	13/39
Type of allergy (IgE/non-IgE)	10/10	10/10	–	3/4	7/6
Type of allergy† (skin/food/respiratory)	18/2/2	18/2/2	–	7/1/0	11/2/1
Age onset allergy (days)	–	126.5 [69, 299]	–	–	–
Mode of delivery (vaginal/cesarean)	52/20 ‡	13/7	39/13	–	–
Age (days)					
baseline	41.5 [1–111]	23 [1–111]	52.5 [2–111]	53 [2–108]	37 [1–111]
6 months	180 [166–227]	179 [166–192]	180 [167–227]	180 [168–227]	180 [166–206]
12 months	364 [345–383]	365.5 [348–383]	363.5 [345–378]	365 [345–377]	363.5 [348–383]
Breastfeeding (yes/no)					
baseline	72/0	20/0	52/0	20/0	52/0
6 months	71/1	20/0	51/1	20/0	51/1
12 months	58/14	17/3	41/11	18/2	40/12
Formula feeding (yes/no)					
baseline	0/72	0/20	0/52	0/20	0/52
6 months	7/65	2/18	5/47	1/19	6/46
12 months	23/49	6/14	17/35	4/16	19/33
Milk feeding§ (BF/FF/MMF)					
baseline	72/0/0	20/0/0	52/0/0	20/0/0	52/0/0
6 months	65/1/6	18/0/2	47/1/4	19/0/1	46/1/5
12 months	49/12/11	12/2/4	35/10/7	48/2/2	33/10/9
Complementary feeding (yes/no)					
baseline	0/72	0/20	0/52	0/20	0/52
6 months	55/17	13/7	42/10	15/5	40/12
12 months	72/0	20/0	52/0	20/0	52/0

†The two subjects who had developed IgE-mediated food/respiratory allergy were also diagnosed with IgE-mediated skin allergy

‡Even though the numbers for allergy and delivery mode are the same (20/52), the infants in the four groups are different. More specifically 39 non-allergic and 13 allergic subjects were delivered vaginally; while 13 were non-allergic and delivered via a C-section; 7 were allergic and delivered via a C-section

§Age reflects the age at the time of fecal sample collection

¶*BF* breastfed, infants receiving breast milk and no formula milk, *FF* formula-fed, infants receiving infant formula milk and not breast milk, *MMF* mixed milk-fed – infants receiving breast milk and formula milk

Numeric variables are presented as median [range]; categorical variables are presented as numbers of participants. Additional clinical characteristics are summarized in Table S7

### Age has a significant impact on the fecal metabolome

To explore the impact of age, diet, delivery mode, and allergy on the fecal metabolome, a range of host and gut microbial metabolites, including (aromatic) amino acids (AAs) and derivatives, B vitamins, nucleobases, nucleosides, BAs, LCFAs, and SCFAs were examined (Table S2).

**Fig. 1:**
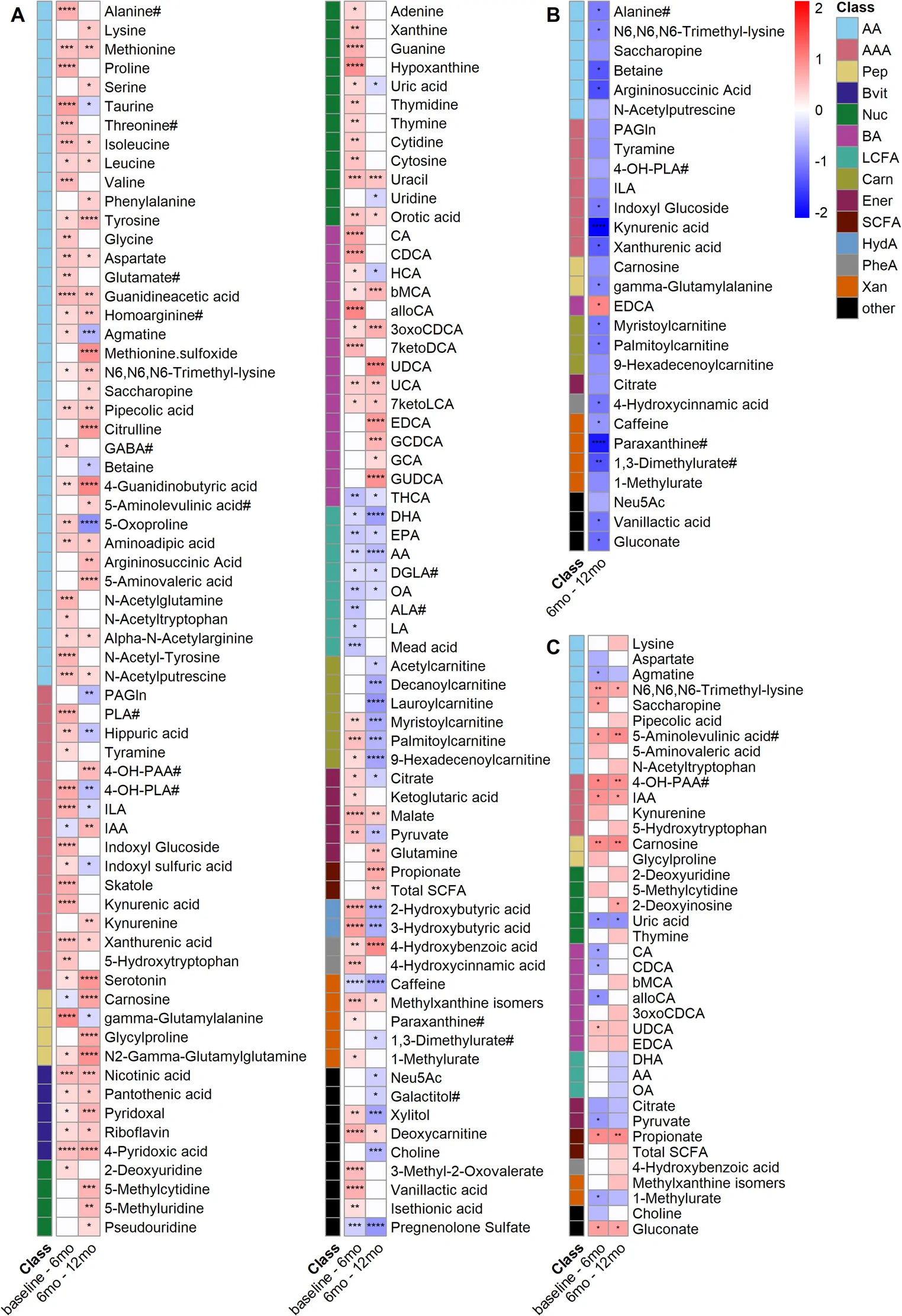
Fecal metabolome alterations associated with age **A**, cessation of breastfeeding **B**, introduction of complementary feeding **C** between baseline and 6mo and/or 6mo and 12mo, assessed using LMM. Colors represent the model coefficient: positive (red), negative (blue), *P* > 0.05 (white). In **A** a positive coefficient represents an increase of the metabolite between the visits; in **B** a positive coefficient represents an increase of the metabolite associated with cessation of breastfeeding between 6mo and 12mo; while in **C** a positive coefficient represents an increase of the metabolite with introduction of complementary feeding between baseline and 6 m or between 6mo and 12mo. Metabolite class annotation: AA - amino acids and derivatives; AAA - aromatic amino acid metabolites; Pep - dipeptides and tripeptides; Bvit - B vitamins and derivatives; Nuc - nucleobases, nucleosides and derivatives; BA - bile acids; LCFA - long-chain fatty acids; Carn - carnitines; Ener - energy metabolites; SCFA - short-chain fatty acids; HydA - hydroxy acids and derivatives; PheA - phenolic acids; Xan – xanthines. Asterisks indicate statistical significance: Q < 0.1 (*), Q < 0.01 (**), Q < 0.001 (***), Q < 0.0001 (****). The “#” in the metabolite names indicates that the metabolite coeluted with another target metabolite. All abbreviations and coeluting metabolites can be found in Table S2

Age and the accompanying dietary changes had a strong effect on the metabolome, as revealed by principal component analysis (PCA) (Figure S2). LMM analysis identified 99 metabolites that significantly changed within the first 6 months of life and 92 metabolites in the second half of the first year (Fig. 1A, Table S8). B vitamins and derivatives, AAs and derivatives, BAs, nucleobases, nucleosides and derivatives, SCFAs, and phenolic acids increased significantly throughout the whole first year, between baseline and 6mo or between 6mo and 12mo. Among those the primary BAs, CA and CDCA, increased in the first six months, glyco-conjugated BAs in the latter six months, and secondary BAs during either or both halves of the year (Fig. 1A).

Host tryptophan metabolites also increased with age, whereas the microbial aromatic AA metabolites followed varying time trends. Aromatic lactic acids (PLA, ILA, 4-OH-PLA#) increased until 6mo. Then, while PLA remained unchanged, ILA and 4-OH-PLA# decreased. Meanwhile, the acetic aromatic acids 4-OH-PAA# and IAA increased after 6mo, with IAA decreasing before 6mo (Fig. 1A).

LCFAs declined through the first year, except for ALA#, LA, and mead acid which declined significantly only until 6mo. Even though an overall decline in acylcarnitines was observed after 6mo, before 6mo the long-chain acylcarnitines increased, whereas the short- and medium-chain acylcarnitines remained unchanged (Fig. 1A).

The metabolites that could not be analyzed using LMMs were assessed using a Mann-Whitney U test (Figure S3A). Consistent with LMM findings, acylcarnitines decreased, whereas AAs and derivatives; B vitamins and derivatives; nucleobases, nucleosides and derivatives; SCFAs; phenolic acids; and host tryptophan metabolites increased over time. The acetic and propionic aromatic acids, PAA and 4-OH-PPA, also increased whereas IPA levels remained stable despite the decline in its missingness with age (Table S4). TUDCA and secondary BAs also increased, particularly in the second half of the first year (Figure S3A). Although LCA and DCA did not pass QC, visual inspection suggested a rise, especially in some subjects at 12mo (Figure S4).

### Dietary changes were associated with fecal metabolome alterations

Infant diets evolved during the first year (Table 1), where at baseline (< 16 weeks), all infants were breastfed, at 6mo 90% infants were breastfed, 8.3% mixed-milk-fed (breastfed and formula-fed), and 1.4% formula-fed, while at 12mo, 68% were breastfed, 15% mixed-milk-fed, and 17% formula-fed. Meanwhile, complementary feeding had started for 76% of the participants by 6mo and for all by 12mo.

Initiation of formula-feeding had a minor effect on the metabolome (LMM, Table S8). It was associated with lower levels of B vitamins i.e. pyridoxal, pantothenic acid, nicotinic acid as well as thymine, 2-deoxyuridine, 2-deoxyinosine but with higher guanosine# and allantoin until 6mo. However, following multiple testing correction, only the association of thymine remained significant.

Complementary feeding was associated with significantly higher propionate, carnosine, and aromatic acetic acids but lower pyruvate levels among others (Table S8, Fig. 1C). Until 6mo, complementary feeding was also negatively associated with the primary BAs but positively with the secondary BA UDCA and syringic acid. The latter was detected only after the introduction of complementary food except for one infant (Figure S5).

Meanwhile, the cessation of breastfeeding was associated with higher EDCA but lower long-chain acylcarnitines, caffeine and metabolites, Neu5Ac, gamma-glutamylalanine, and 4-hydroxycinnamic acid. The tryptophan and tyrosine metabolites kynurenic acid, indoxyl glucoside, xanthurenic acid (Q < 0.1), ILA, tyramine, and 4-OH-PLA# (0.01 < *P* < 0.05, Q > 0.1) were also negatively associated with cessation of breastfeeding (Fig. 1B).

The effect of feeding practices for metabolites that could not be analyzed using LMM analysis, were assessed using the Mann-Whitney U test (Figure S3B, C, Table S9). Butyrate, secondary BAs, and phenolic acids were higher in the complementary-fed versus non-complementary-fed infants at 6mo (Q < 0.1) and breastfed versus non-breastfed subjects at 12mo (*P* < 0.05). N2,N2-dimethylguanosine and 2-octenoylcarnitine were respectively higher and lower in the complementary-fed versus non-complementary-fed infants, whereas the tryptophan metabolite IPA was higher in the non-breastfed versus breastfed infants.

### Delivery mode affected the fecal metabolome up to 6 months of age

**Fig. 2:**
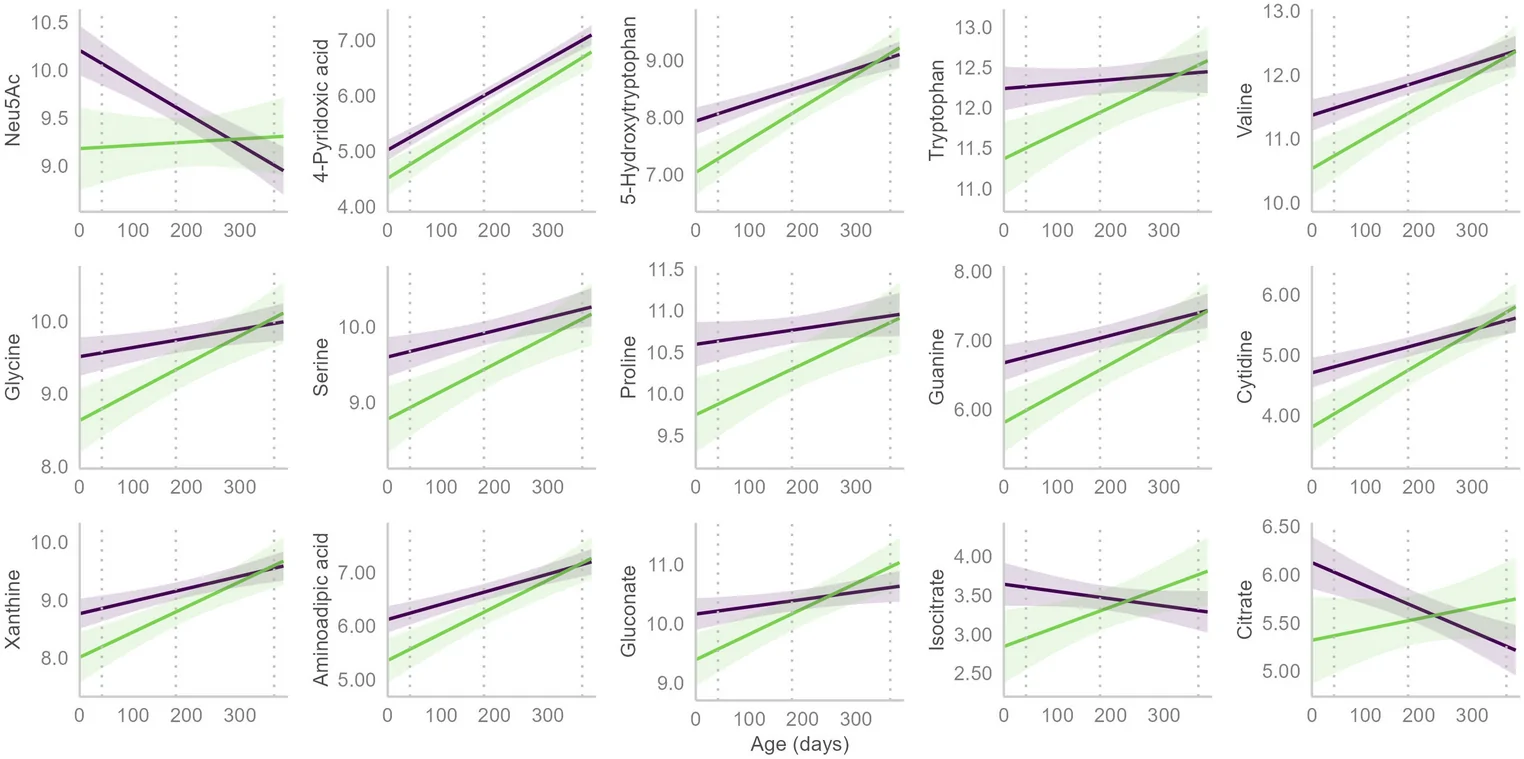
Scaled abundance levels of the metabolites that significantly differed between infants delivered vaginally (purple, solid) and via C-section (green, solid) group as a function of age, based on LMM analysis. The shaded areas represent the 95% confidence intervals, while the dotted grey lines represent the median age at each visit (baseline, 6mo, 12mo). All abbreviations and coeluting metabolites can be found in Table S2

TEMPO enrolled infants delivered vaginally and via a C-section, allowing an investigation into the effect of the delivery mode on the metabolome. Fifteen metabolites, including Neu5Ac, AAs and derivatives, pyrimidine and purine derivatives, and carboxylic acids, were significantly lower in the C-section compared to the vaginal group at baseline (Fig. 2, Table S10). For all, the group differences decreased with age until the groups completely overlapped at 12mo. Notably, Neu5Ac levels remained stable over time in the C-section group, while they declined in the vaginal group. In contrast, proline and tryptophan were stable in the vaginal group but increased over time in the C-section group. Citrate and isocitrate also followed opposing trends, decreasing in the vaginal group while increasing in the C-section group.

### Higher LCFA levels in infants who developed allergy

**Fig. 3:**
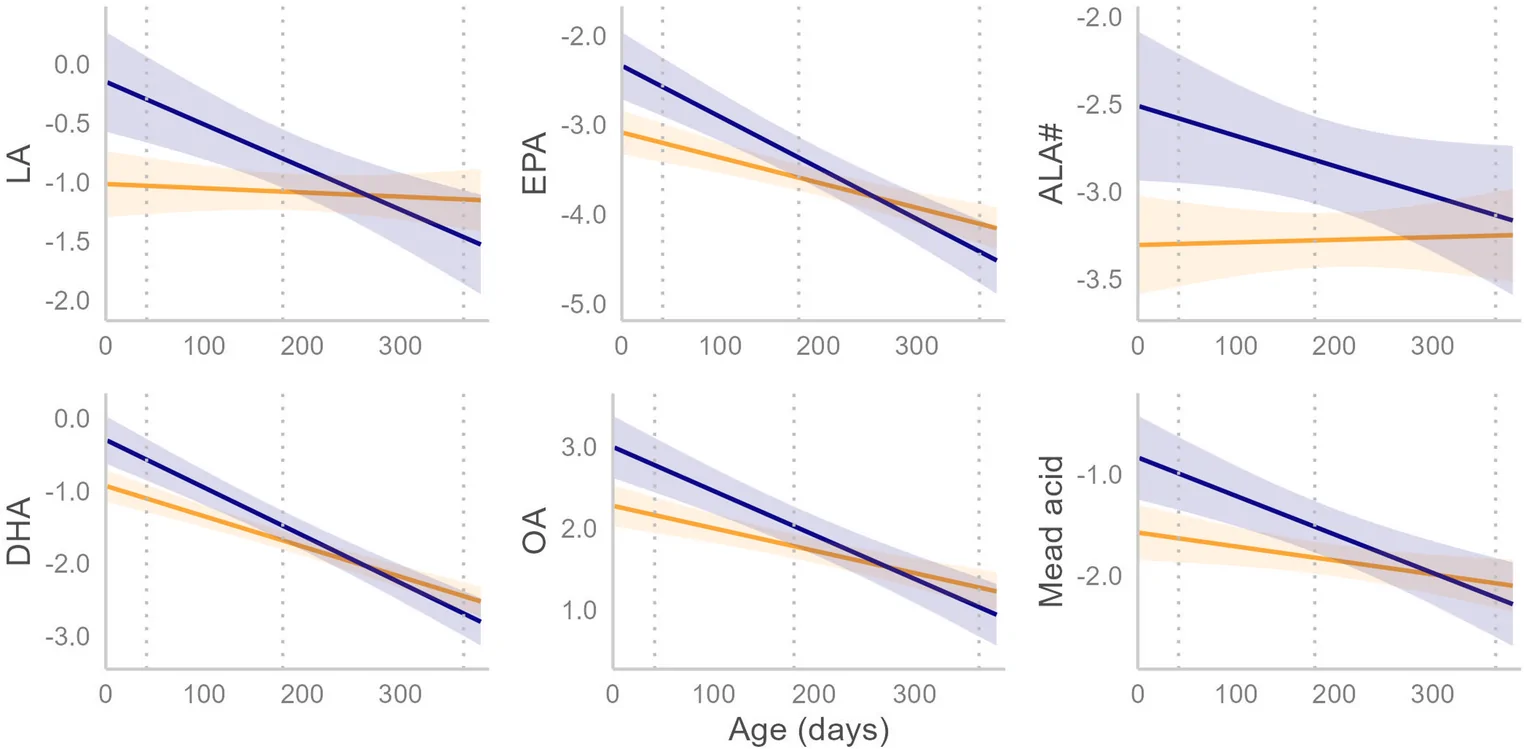
Scaled relative abundance levels of LCFAs as a function of age in allergic (blue, solid) and non-allergic (orange, solid) groups, based on LMM analysis. The shaded areas represent the 95% confidence intervals, while the dotted grey lines represent the median age at each visit (baseline, 6mo, 12mo). The “#” in the metabolite names indicates that the metabolite coeluted with another target metabolite. All abbreviations and coeluting metabolites can be found in Table S2

LMMs were used to examine the longitudinal metabolite alterations with age between the infants who developed allergies during the first year of life and those who did not. At baseline, no participants were allergic, and allergies developed between 69 and 299 days (median age 126.5 days). A few LCFAs, namely LA, EPA, ALA#, DHA, OA, and mead acid, were found to be significantly higher at baseline in the allergic compared to the non-allergic group (Table S11). However, the group separation disappeared over time and the groups overlapped at 6mo and 12mo (Fig. 3). In LMM that stratified the allergy group by allergy type, no group differences reached significance after multiple testing correction. However, when comparing these results to those from the original model, the observed elevations in LCFA levels appear to be primarily driven by the non-IgE-mediated group, as several LCFAs rank among the metabolites with the lowest P-values (Table S12). In the IgE-mediated group, LCFAs remain prominent, but additional metabolites-such as deoxycarnitine, cadaverine, and ornithine-also emerge as top hits (Table S12). A subsequent linear model analyses of the baseline samples, comparing the allergic and non-allergic infants, did not reveal any significant differences between the groups (Table S13), possibly due to the reduced statistical power of the model. Comparable analysis of IgE‑mediated and non‑IgE‑mediated infants at baseline also showed no evidence of group differences (Table S14).

### Lower *Bifidobacterium* spp. in infants prior to allergy development

**Fig. 4:**
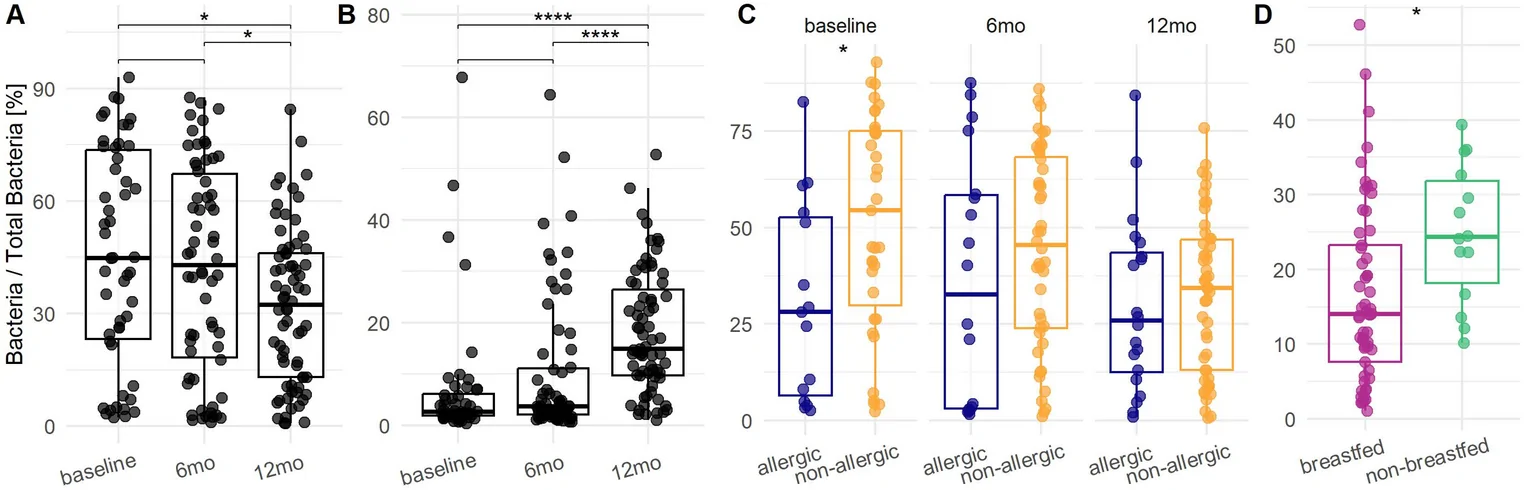
The levels of **A** Bifidobacterium spp. between visits; **B** ER/CC between visits; **C ***Bifidobacterium* spp. between the allergic (blue) and non-allergic (orange) infants at each visit; **D** ER/CC between breastfed (pink) and non-breasted (green) at 12mo as proportion of the total bacteria. Statistical analysis was performed using Mann-Whitney test: *P* < 0.05 (*), *P* < 0.01 (**), *P* < 0.001 (***), *P* < 0.0001 (****), for Q values refer to Table S10. Number of measurements per group and visit: *Bifidobacterium* spp.: n = [50, 62, 70]; ER/CC: n = [48, 60, 71] for baseline, 6mo and 12mo, respectively

FISH was applied to quantify the *Bifidobacterium* spp. which are characteristic GM members in breastfed infants and ER/CC, which is primarily composed of *Lachnospiraceae* species and is more common in adults (Mueller et al., 2006). The analysis showed that ER/CC levels were significantly higher at 12mo compared to baseline and 6mo, whereas the opposite was the case for *Bifidobacterium* spp. (*P* < 0.05, Q < 0.1, Fig. 4A, B). The decline in *Bifidobacterium* spp. from 6mo to 12mo was also found to be positively associated with the decline in ILA and 4-OH-PLA# (0.39 < ρ < 0.43; *P* < 0.05, Q > 0.1, Table S15). ER/CC was also significantly lower in infants that were still breastfed versus non-breastfed infants (*P* < 0.05, Q < 0.1, Fig. 4D) and those not receiving formula versus those that did at 12mo (*P* < 0.05, Q > 0.1, Figure S6). Complementary feeding and delivery mode were not associated with significant differences in the examined taxa (Figure S6). The baseline *Bifidobacterium* spp. levels of the infants who developed allergy by 12mo were lower than those that did not (*P* < 0.05, Q > 0.01, Fig. 4C). A follow-up Spearman correlation analysis showed no evidence of a correlation between reduced *Bifidobacterium* spp. levels and elevated LCFAs levels (Figure S7).

## Discussion

In this study, healthy breastfed infants at risk of allergy were followed throughout their first year. During this period, alteration in the fecal metabolome and key microbiome members (*Bifidobacterium* spp., ER/CC) were examined in relation to age, feeding practices, mode of delivery, and allergy development. Strong age-associated alterations were observed including an overall increase in AAs and derivatives, BAs, nucleobases, nucleosides and derivatives, B vitamins and derivatives, SCFAs, and phenolic acids, along with a decrease in LCFAs and acylcarnitines. Feeding practices, specifically the intake of complementary food and cessation of breastfeeding were significantly associated with changes to the metabolome. Delivery mode had a pronounced impact on the metabolome with distinct differences observed mainly at baseline, some of which persisted until 6mo. Meanwhile, infants who developed allergy had lower *Bifidobacterium* spp. and significantly higher LCFA levels at baseline.

These strong age-associated metabolome changes align with previous studies examining the fecal metabolome in early life (Holzhausen et al., 2023; Ouyang et al., 2023). These pronounced shifts are expected, given the rapid physical growth (Yatsunenko et al., 2012) and GM diversification associated with the transition from milk to solid food during this period (Laursen et al., 2016). In our cohort, the diversification is evident by the significant decline in bifidobacteria and the increase in the adult-like ER/CC at 12mo as well as higher ER/CC in non-breastfed versus breastfed infants at 12mo.

The introduction of complementary feeding from around 6mo, likely accompanied by a shift toward greater dietary diversity and increased fiber and protein intake, and the associated GM diversification, (Laursen et al., 2016) are also clearly reflected at the metabolomic level. The decline in pyruvate after 6mo following an initial increase, and its negative association with complementary feeding, likely reflects its conversion to downstream metabolites as the GM diversifies (Roager et al., 2023). The increase in fiber intake was also evident by the rise of butyrate and propionate after 6mo and their positive association with complementary feeding and cessation of breastfeeding, respectively, in agreement with Tsukuda et al. (Tsukuda et al., 2021). That aligns well with the observed increase in the well-known butyrate producers within ER/CC (Barcenilla et al., 2000). The observed temporal increase in phenolic acids, alongside associations with breastfeeding and complementary feeding, is consistent with their diverse origins, including plants (Kiriyama et al., 2024), breast milk (Fiecke et al., 2025), and microbial flavonoid and tyrosine transformation (Kiriyama et al., 2024). Meanwhile, the higher levels of carnosine (Cuparencu et al., 2024; FooDB, n.d.; Mitry et al., 2019) and N2,N2-dimethylguanosine (FooDB, n.d.; Wishart et al., 2022) in complementary-fed infants may indicate meat consumption.

In this work, A transition from a bifidobacteria‑dominated microbiome to a more adult‑like community was also reflected in changes in microbial aromatic amino acid metabolism, shifting from the production of aromatic lactic acids toward aromatic acetic and propionic acids. This pattern aligns with the sequential rise in fecal aromatic lactic, acetic, and propionic acids during the first six months of life previously reported (Laursen et al., 2023). Aromatic lactic acids (ILA, 4-OH-PLA#, PLA#), produced by infant-type bifidobacteria, (Laursen et al., 2021) increased until 6mo, after which ILA and 4‑OH‑PLA# declined, in agreement with previous findings (Sillner et al., 2021). As expected, the decrease in these aromatic lactic acids correlated positively with the decline in *Bifidobacterium* spp. observed at the same period. Meanwhile, the aromatic acetic and propionic acids (IAA, 4-OH-PAA, PAA, 4-OH-PPA) increased after 6mo and were positively associated with complementary feeding, likely reflecting increased microbial protein degradation (Roager et al., 2023).

As anticipated, the abundance and diversity of secondary BAs increased with age and the two drivers of GM diversification: introduction to complementary foods and the cessation of breastfeeding. Similar to Sillner et al. (2021) we report on less-studied secondary BAs (7ketoLCA, 3oxoCDCA, 7,12oxoLCA, 7oxoDCA, 3oxoCA, UCA) in infancy, along with almost complete absence of LCA and DCA until 12mo (Sillner et al., 2021). The latter aligns with the observed increase in ER/CC well-known for its high 7α-dehydroxylating activity required for their production (Ridlon et al., 2014). Meanwhile, the rise in glyco-BAs after 6mo likely reflects the reduction in particularly effective glyco-BAs deconjugators bifidobacteria (Kim et al., 2004). Even though B vitamins are obtained from the diet, including breast milk (Qiao et al., 2022), their temporal increase observed in this study is likely attributed to increased microbial production (Wan et al., 2022) as the GM’s function diversifies and more B vitamin-producing microbes become established.

The decline in acylcarnitines after 6mo and their positive association with breastfeeding, along with the negative association of LCFAs with age suggest increasing reliance on beta oxidation by the host. Production of conjugated linoleic and linolenic acid isomers by bifidobacteria (Gorissen et al., 2010) possibly also contributes to the decline in LA and ALA# before 6 m, a period characterized by bifidobacterial dominance.

Multiple studies have shown strong fecal metabolome differences between breastfed and formula-fed infants (Chalifour et al., 2023; He et al., 2019; Holzhausen et al., 2023; Sillner et al., 2021). However, unlike these studies, our cohort consisted of infants breastfed for at least 16 weeks, with formula-feeding often initiated alongside breastfeeding, mainly after the introduction of complementary feeding. The minor significant associations observed with formula feeding in this cohort (a single significant metabolite following multiple testing correction), agree with He et al. (2019) who reported convergence of the metabolome profiles between breast-fed and formula-fed infants following complementary feeding (He et al., 2019).

Despite its known importance in shaping the GM (Rutayisire et al., 2016), delivery mode was not associated with microbiome differences in this cohort, most likely due to the limited scope of our FISH analysis. It did, however, affect the metabolome, especially at baseline and up to 6mo. Earlier studies reported no metabolome changes despite shifts in the microbiome composition (Hoen et al., 2021), or significant alterations that differ from our findings and between each other (Li et al., 2021; Ouyang et al., 2023). These discrepancies may reflect ethnic or age-related cohort differences. We found the HMO building-block Neu5Ac to be significantly higher in the vaginal compared to the C-section group and maternal pre-pregnancy BMI to be associated with C-section. Since pre-pregnancy BMI has been linked to HMO composition (Han et al., 2021), the difference in Neu5Ac may be attributed to variations in breast milk composition. Another possibility is that vaginally-delivered infants’ guts are richer in taxa like *Bacteroides* capable of cleaving sialic acids HMO residues (Kijner et al., 2024).

Unexpectedly, no metabolome differences were observed between the allergic groups at 6mo and 12mo, despite the emergence of allergic symptoms in this period. Feeding changes and the resulting GM shifts may have masked these differences. Infants who developed allergy during the study did, however, have significantly higher baseline levels of the LCFAs, mainly polyunsaturated fatty acids (PUFAs), including n-6 LA and n-3 EPA, ALA#, DHA. Although elevated plasma n-3 and n-6 PUFA levels have also been reported in children with food allergy (Crestani et al., 2024), lower n-3 PUFA levels are generally associated with increased allergy risk (Jonsson et al., 2016; Lee-Sarwar et al., 2019). Along with the higher LCFA levels, we observed lower *Bifidobacterium* spp. in the allergic group. The absence of significant correlation between the two, however, suggests that the lower LCFA levels in the allergic infants are unlikely to be due to bifidobacteria. Instead, the difference may be due to variations in mother’s breast milk composition (Bobiński & Bobińska, 2020), microbial transformation (Gorissen et al., 2010), or differences in intestinal absorption, necessitating further investigation. The low bifidobacterial levels observed are in agreement with reports of consistently reduced bifidobacterial levels in children with cow’s milk allergy (Savova et al., 2024) and on the importance of early *Bifidobacterium* spp. colonization for immune regulation (Henrick et al., 2021). Interestingly, the decline in bifidobacteria was not accompanied by reduced levels of immune-regulatory metabolites. This included SCFAs, which have been associated with allergy development (Szukalska et al., 2025), as well as bifidobacterial aromatic lactic acids: ILA (Laursen et al., 2021) and 4-OH-PLA, the latter of which has been recently associated with IgE-mediated food allergy sensitization (Myers et al., 2026).

Our study has several limitations, including the small sample size, the wide age range at baseline, and the infrequent sampling. The limited sample size, especially in the allergic group, prevented a separate analysis of the different allergy types. Moreover, the microbiome analysis by means of FISH was limited to only two major taxonomic groups of infant gut microbiome (Laursen et al., 2016). To enhance metabolomic interpretation and clarify the GM–allergy link, future research should consider whole microbiome dynamics using 16S rRNA-gene or metagenomic sequencing rather than focusing solely on specific taxa and prioritize integrated microbiome–metabolome analyses. Meanwhile, examining the circulating metabolome and breast milk compositional analysis are of interest to respectively understand the plausible link between LCFAs and allergy and aid the interpretation of the delivery mode findings.

This study offers valuable new insights into the longitudinal fecal metabolome development in infancy, a critical period with lasting implications for immune system development. Our findings reveal substantial metabolomic shifts with age likely due to changes to the host metabolism, diet, and the GM. Notably, we show that C-section is significantly associated with fecal metabolome alterations up to 6mo, though the health implications of these changes require further investigation. This study showed that low *Bifidobacterium* spp. and LCFAs precede allergy, indicating a temporal association that suggests a direction for follow-up studies on their potential role in allergy development.

## Supplementary Information

Below is the link to the electronic supplementary material.

Supplementary Material 1

Supplementary Material 2

## Data Availability

The metabolomics data of this study are submitted in MetaboLights at http://www.ebi.ac.uk/metabolights/with reference number MTBLS12775, along with limited clinical metadata. Additional individual-level metadata, even pseudonymized, are sensitive and are protected by the GDPR and not publicly available. Reasonable data sharing requests based on data processing and material transfer agreements can be made to Danone Research & Innovation ([https://www.danoneresearch.com/](https:/www.danoneresearch.com)).

## Abbreviations

HMO: Human milk oligosaccharide
FISH: Fluorescence in situ hybridization
ER/CC: Eubacterium rectale/Clostridium coccoides
GC-FID: Gas chromatography coupled - flame ionization detector
LC-MS: Liquid chromatography – mass spectrometry
LMM: Linear mixed model
AA: Amino acid
PUFA: Polyunsaturated fatty acid
