# Supplementary material for: Fecal metabolome alterations in infants at risk of developing allergies during the first year of life
Source: Metabolomics. 2026 Jul 1;22(4):112. doi: 10.1007/s11306-026-02478-6 (PMC13323787; doi:10.1007/s11306-026-02478-6)
Supplement: Supplementary file 2 — Supplementary Material 2 [file 11306_2026_2478_MOESM2_ESM.docx]

**Supplementary information**

**Fecal metabolome alterations during the first year of life in infants at risk for allergies**

Authors: Mariyana V. Savova^1^, Pingping Zhu^1^, Alida Kindt^1^, the TEMPO study team, Harm Wopereis^2^, Clara Belzer^3^, Amy C. Harms^1*^, Thomas Hankemeier^1^

^1^Metabolomics and Analytics Centre, Leiden Academic Centre for Drug Research, Leiden University, Leiden 2333 CC, the Netherlands

^2^Danone Research & Innovation, Uppsalalaan 12, 3584 CT Utrecht, the Netherlands

^3^Laboratory of Microbiology, Wageningen University, Stippeneng 4, 6708 WE Wageningen, the Netherlands

^*^Corresponding author: Dr. Amy C. Harms, Metabolomics and Analytics Centre, Leiden Academic Centre for Drug Research, Leiden University, Leiden 2333 CC, Netherlands

E-mail: a.c.harms@lacdr.leidenuniv.nl

**Supplementary methods**

**Data analysis details**

Two LMMs were built to investigate the effect of age and diet on the fecal metabolome with age (days), breastfeeding (yes/no), formula feeding (yes/no), and complementary feeding (yes/no) used as fixed effects and subject ID as a random effect. The first model investigated the baseline and 6m measurements (*Metabolite ~ age + complementary feeding + formula feeding + (1|ID)*), whereas the second the 6m and 12m measurements (*Metabolite ~ age + complementary feeding + formula feeding + breastfeeding + (1|ID)*). Reference levels for the variables were the median age at baseline, “no” for complementary and formula feeding, and “yes” for breastfeeding. Breastfeeding was included only in the second model since a single subject was non-breastfed at 6m (Table 1). As infants’ diet changed during the first year (Table 1), the feeding practices (breastfeeding, formula feeding, complementary feeding) at each visit were considered. Because of the change in feeding practices and due to the choice of reference levels, the breastfeeding, complementary feeding and formula feeding coefficients are interpreted respectively as cessation of breastfeeding, introduction of complementary feeding and introduction of formula feeding. To study the effect of delivery mode on the metabolome a LMM with age (days), delivery mode (Vaginal/C-section), and their interaction as fixed effects was used (*Metabolite ~ age + delivery mode + age:delivery mode + (1|ID)*). Reference levels for the variables were the median age at baseline and “Vaginal” delivery. Similarly, the LMM for allergy had age, allergy status at 12 months (allergic, non-allergic) and their interaction as fixed effects (*Metabolite ~ age + allergy status + age:allergy status + (1|ID)*). Median age at baseline and the non-allergic group were used as a reference. A separate LMM was then constructed after stratifying the allergy group by allergy type (IgE-mediated, non-IgE–mediated, and non-allergic), with the non-allergic group as the reference. Before building any of the LMMs the input data was scaled by the standard deviation of all samples. A sub-analyses of the baseline samples were carried out using the following linear model Metabolite ~ age + allergy status + age: allergy status. One of the analyses examined the allergic and non-allergic infants with non-allergic group as reference, while the other the IgE-mediated and non-IgE mediated infants with the IgE-mediated group as a reference.

We used R v. 4.3.2 (R Core Team 2023a), rmarkdown v. 2.29 (Xie, Allaire, and Grolemund 2018; Xie, Dervieux, and Riederer 2020; Allaire et al. 2024), and the following R packages: car v. 3.1.3 (Fox and Weisberg 2019), ggpubr v. 0.6.0 (Kassambara 2023), grid v. 4.3.2 (R Core Team 2023b), gridExtra v. 2.3 (Auguie 2017), imputeLCMD v. 2.1 (Lazar and Burger 2022), knitr v. 1.45 (Xie 2014, 2015, 2023), lmerTest v. 3.1.3 (Kuznetsova, Brockhoff, and Christensen 2017), openxlsx v. 4.2.5.2 (Schauberger and Walker 2023), pheatmap v. 1.0.12 (Kolde 2019), tidyverse v. 2.0.0 (Wickham et al. 2019), writexl v. 1.5.1 (Ooms 2024). The package grateful v. 0.3.0 was used to generate the citation of the used packages Rodriguez-Sanchez F, Jackson C (2025).

**Package citations**

Allaire, JJ, Yihui Xie, Christophe Dervieux, Jonathan McPherson, Javier Luraschi, Kevin Ushey, Aron Atkins, et al. 2024. rmarkdown: Dynamic Documents for r. https://github.com/rstudio/rmarkdown.

Auguie, Baptiste. 2017. gridExtra: Miscellaneous Functions for “Grid” Graphics. https://CRAN.R-project.org/package=gridExtra.

Fox, John, and Sanford Weisberg. 2019. An R Companion to Applied Regression. Third. Thousand Oaks CA: Sage. https://www.john-fox.ca/Companion/.

Kassambara, Alboukadel. 2023. ggpubr: “ggplot2” Based Publication Ready Plots. https://CRAN.R-project.org/package=ggpubr.

Kolde, Raivo. 2019. pheatmap: Pretty Heatmaps. https://CRAN.R-project.org/package=pheatmap.

Kuznetsova, Alexandra, Per B. Brockhoff, and Rune H. B. Christensen. 2017. “lmerTest Package: Tests in Linear Mixed Effects Models.” Journal of Statistical Software 82 (13): 1–26. https://doi.org/10.18637/jss.v082.i13.

Lazar, Cosmin, and Thomas Burger. 2022. imputeLCMD: A Collection of Methods for Left-Censored Missing Data Imputation. https://CRAN.R-project.org/package=imputeLCMD.

Ooms, Jeroen. 2024. writexl: Export Data Frames to Excel “xlsx” Format. https://CRAN.R-project.org/package=writexl.

R Core Team. 2023a. R: A Language and Environment for Statistical Computing. Vienna, Austria: R Foundation for Statistical Computing. https://www.R-project.org/.

———. 2023b. R: A Language and Environment for Statistical Computing. Vienna, Austria: R Foundation for Statistical Computing. https://www.R-project.org/.

Rodriguez-Sanchez F, Jackson C (2025). grateful: Facilitate citation of R packages. https://pakillo.github.io/grateful/.

Schauberger, Philipp, and Alexander Walker. 2023. openxlsx: Read, Write and Edit Xlsx Files. https://CRAN.R-project.org/package=openxlsx.

Wickham, Hadley, Mara Averick, Jennifer Bryan, Winston Chang, Lucy D’Agostino McGowan, Romain François, Garrett Grolemund, et al. 2019. “Welcome to the tidyverse.” Journal of Open Source Software 4 (43): 1686. https://doi.org/10.21105/joss.01686.

Xie, Yihui. 2014. “knitr: A Comprehensive Tool for Reproducible Research in R.” In Implementing Reproducible Computational Research, edited by Victoria Stodden, Friedrich Leisch, and Roger D. Peng. Chapman; Hall/CRC.

———. 2015. Dynamic Documents with R and Knitr. 2nd ed. Boca Raton, Florida: Chapman; Hall/CRC. https://yihui.org/knitr/.

———. 2023. knitr: A General-Purpose Package for Dynamic Report Generation in r. https://yihui.org/knitr/.

Xie, Yihui, J. J. Allaire, and Garrett Grolemund. 2018. R Markdown: The Definitive Guide. Boca Raton, Florida: Chapman; Hall/CRC. https://bookdown.org/yihui/rmarkdown.

Xie, Yihui, Christophe Dervieux, and Emily Riederer. 2020. R Markdown Cookbook. Boca Raton, Florida: Chapman; Hall/CRC. <https://bookdown.org/yihui/rmarkdown-cookbook>.

**Supplementary Figures**


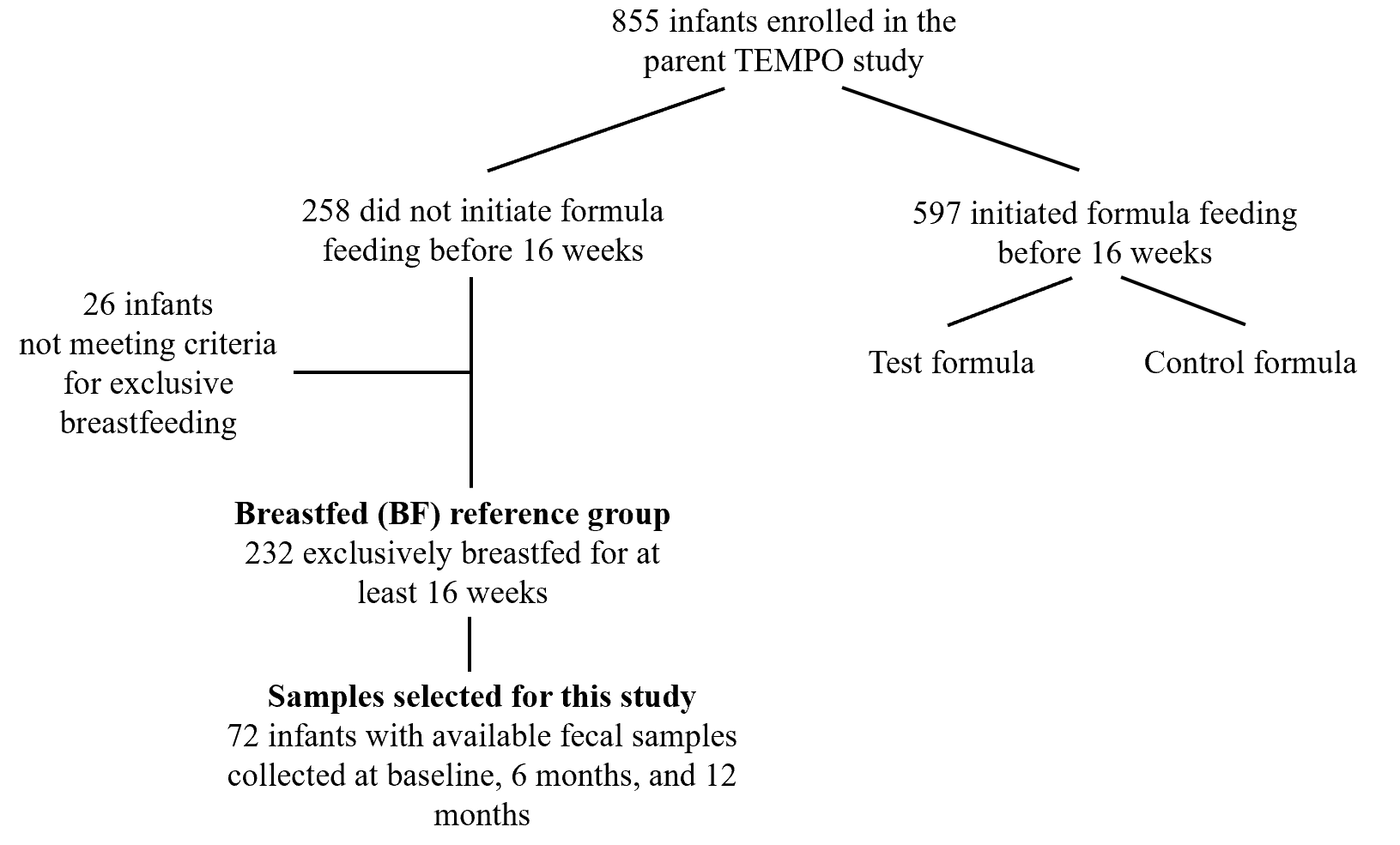


**Figure S1**. Schematic overview of the sample selection process


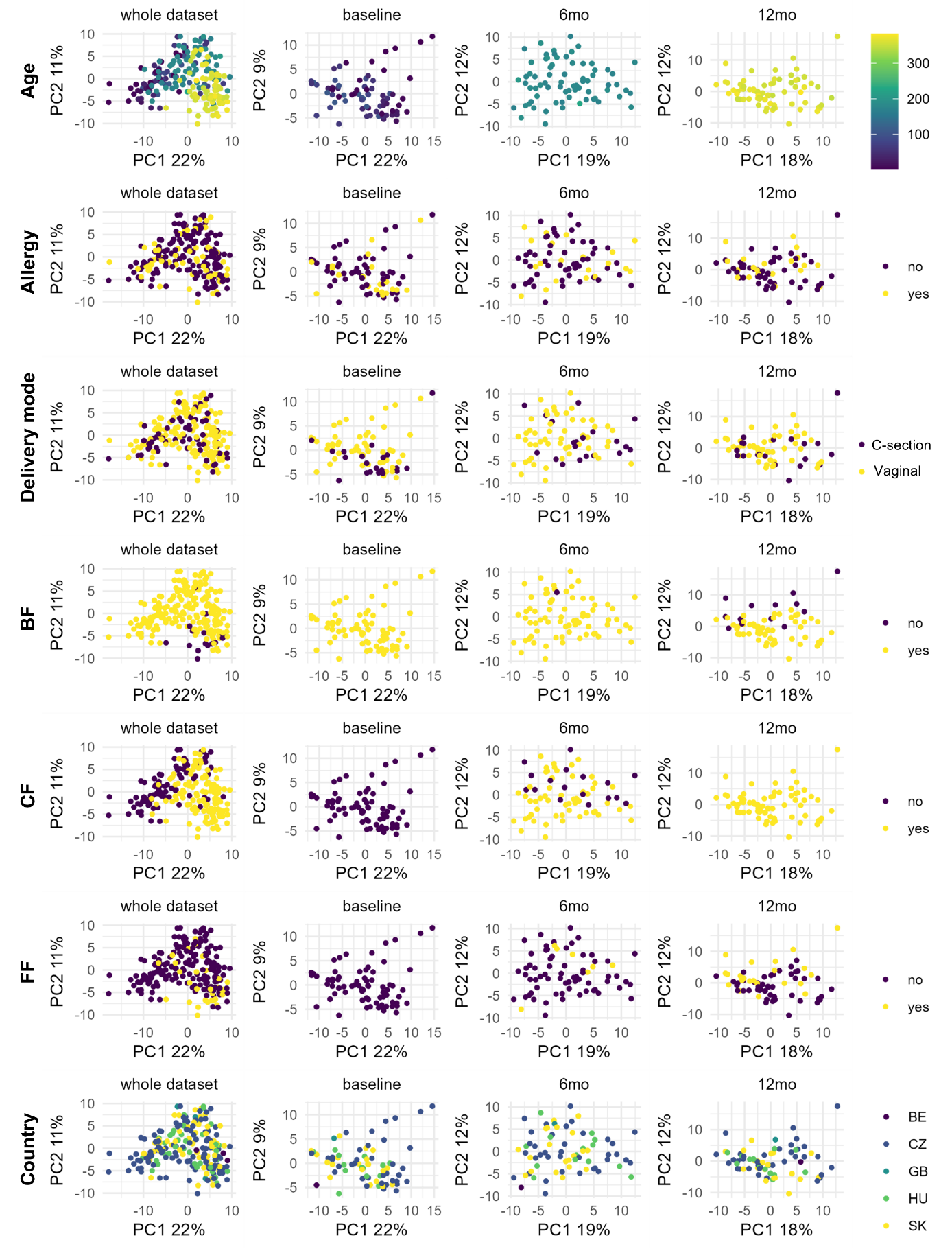


**Figure S2**. Principal Component Analysis (PCA) score plot of the whole dataset and measurements obtained at each visit (baseline, 6months [6mo], 12mo). At each row the datapoints are grouped and coloured according to a different variable: age [day]; allergy (allergy status at 12mo), Delivery mode [C-section, Vaginal], breastfeeding [BF], complementary feeding [CF], formula feeding [FF], country (Belgium [BE], Czechia [CZ], Great Britain [GB], Hungary [HU], Slovakia [SK].


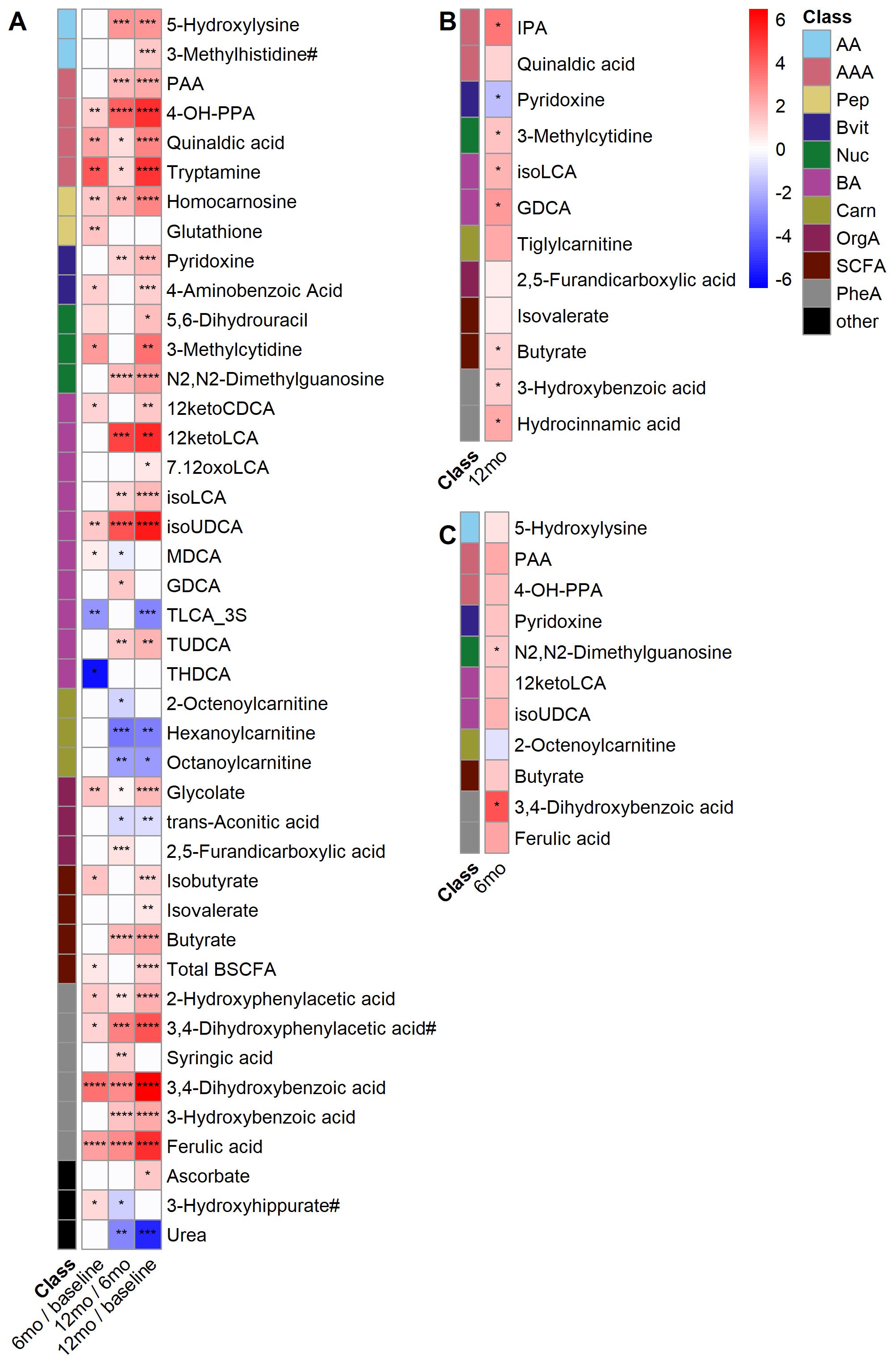


**Figure S3**. Comparison of the fold changes of metabolites A) between the visits , B) breastfeeding groups at 12m, C) complementary feeding at 6m. The comparison was performed using paired Mann-Whitney U test on the metabolites excluded for the LMM analysis due to high missingness. Information on the number of subjects per comparison can be found in Table S10. Colors represent the fold change: above 0 (red), below 0 (blue), p>0.05 (white). Asterisks indicate statistical significance: Q < 0.1 (*), Q < 0.01 (**), Q < 0.001 (***), Q > 0.001 (****). The “#” in the metabolite names indicates that the metabolite has coeluted with another target metabolite. Information on that and all abbreviations can be found in Table S1. Metabolite class annotations: Abbreviations used are as follows: AA - amino acids and derivatives; AAA - aromatic amino acid metabolites; Pep - dipeptides and tripeptides; Bvit - B vitamins and derivatives; Nuc - nucleobases, nucleosides and derivatives; BA - bile acids; Carn - carnitines; OrgA - organic acids; SCFA - short-chain fatty acids; PheA - phenolic acids. The “#” in the metabolite names indicates that the metabolite coeluted with another target metabolite. All abbreviations and coeluting metabolites can be found in Table S1.

| 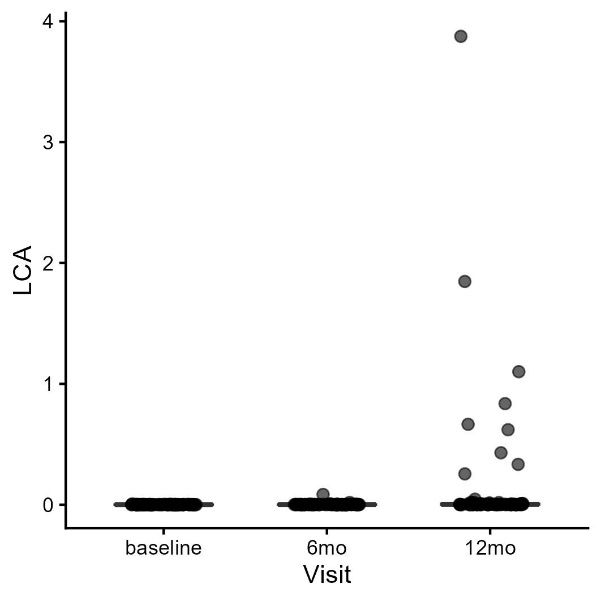 | 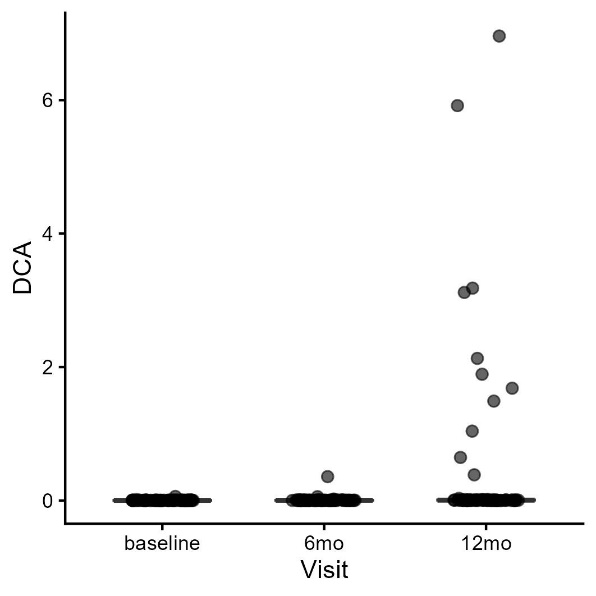 |
| --- | --- |

**Figure S4**. Boxplots showing the distribution of LCA and DCA between the visits (baseline, 6m, 12m). Individual data points are jittered


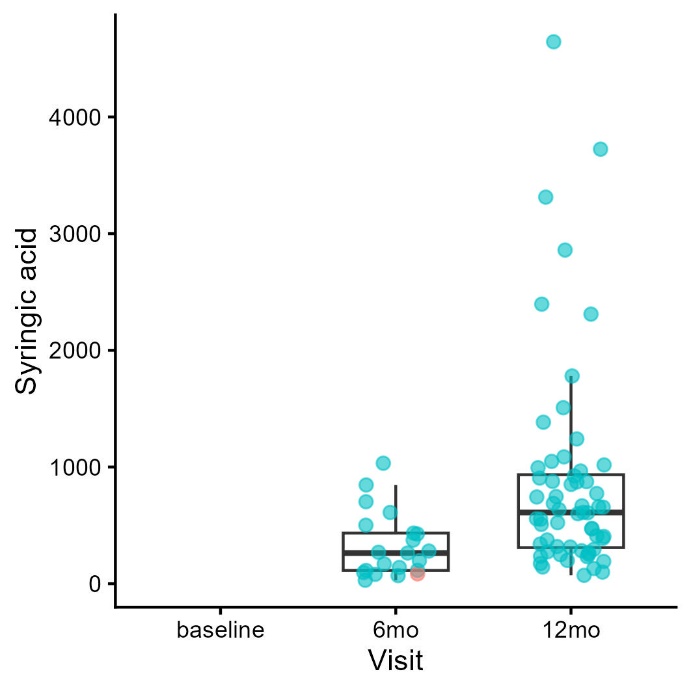


**Figure S5**. Boxplots showing the distribution of syringic acid at the three visits (baseline, 6m, 12m). Individual data points are jittered and colored according to the complementary feeding status: blue – complementary-fed and orange: not complementary fed


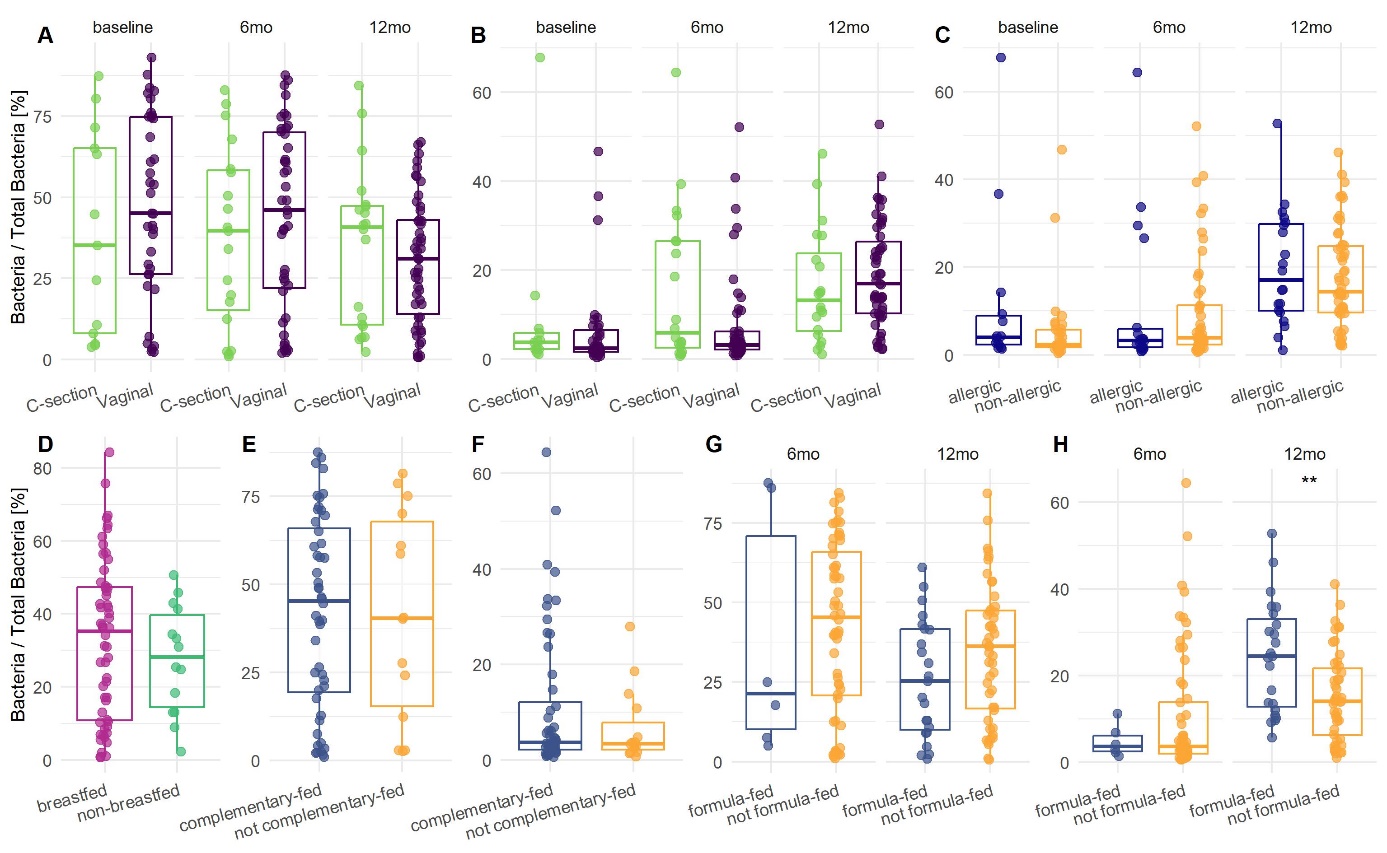


**Figure S6.** Fluorescence *in situ* hybridization (FISH) relative levels of **A)** *Bifidobacterium* spp. between vaginal (purple) and C-section (green) deliveries; **B)** ER/CC between vaginal (purple) and C-section (green) deliveries; **C)** ER/CC between allergic (blue) and non-allergic (orange) infants); **D)** *Bifidobacterium* spp. between breastfed (pink) and non-breasted (green) infants at 12m; **E)** *Bifidobacterium* spp. between complementary-fed (blue) and not complementary-fed (orange) infants at 6m; **F)** ER/CC between complementary-fed (blue) and not complementary-fed (orange) infants at 6m; **G)** *Bifidobacterium* spp. between formula-fed (blue) and not formula-fed (orange) infants; **H)** ER/CC between formula-fed (blue) and not formula-fed (orange) infants. Statistical analysis was performed using Mann-Whitney test: P < 0.05 (*), P < 0.01 (**), P < 0.001 (***), P < 0.0001 (****), for Q values refer to Table S10. Number of measurements per group and visit: *Bifidobacterium* spp.: n = [50, 62, 70]; ER/CC: n = [48, 60, 71] for baseline, 6m and 12m, respectively.


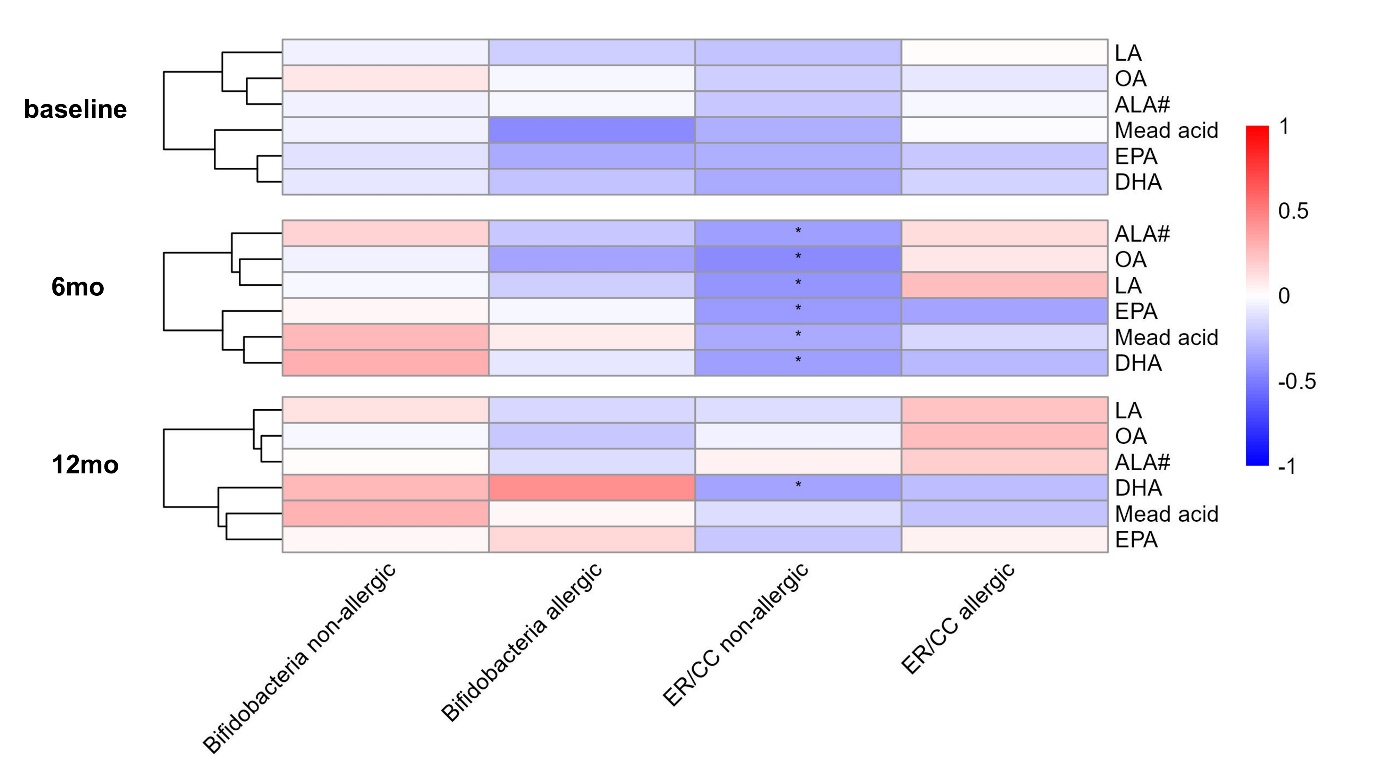


**Figure S7.** Spearman correlation between the long chain fatty acids found to be significantly higher at baseline in the allergic infants and *Bifidobacterium* spp. and ER/CC at baseline, 6m, 12m, Q < 0.1 (*). The “#” in the metabolite names indicates that the metabolite coeluted with another target metabolite. All abbreviations and coeluting metabolites can be found in Table S1.
